# Dilithium 1,2,5-thia­diazo­lidine-3,4-dione 1,1-dioxide dihydrate

**DOI:** 10.1107/S1600536812036379

**Published:** 2012-08-31

**Authors:** Dennis W. McOwen, Samuel A. Delp, Paul D. Boyle, Wesley A. Henderson

**Affiliations:** a Ionic Liquids and Electrolytes for Energy Technologies (ILEET) Laboratory, Department of Chemical and Biomolecular Engineering, North Carolina State University, 911 Partners Way, Raleigh, NC 27695, USA; b X-ray Structural Facility, Department of Chemistry, North Carolina State University, 2620 Yarbrough Drive, Raleigh, NC 27695, USA

## Abstract

The title compound, poly[μ-aqua-aqua-μ_6_-(1,1-dioxo-1λ^6^,2,5-thia­diazo­lidine-3,4-diolato)-dilithium], [Li_2_(C_2_N_2_O_4_S)(H_2_O)_2_]_*n*_ or (H_2_O)_2_:Li_2_TDD, forms an infinite three-dimensional structure containing five-coordinate (Li/5) and six-coordinate (Li/6) Li^+^ cations. Li/5 is coordinated by three water mol­ecules, one carbonyl O atom and one sulfuryl O atom while Li/6 is coordinated by one water mol­ecule, three carbonyl O atoms, and two sulfuryl O atoms. Each water mol­ecule bridges two Li^+^ cations, while also hydrogen bonding to either one endocyclic N atom and one sulfuryl O atom or two endocyclic N atoms. While the endocyclic N atoms in the anion do not coordinate the Li^+^ cations, the carbonyl and sulfuryl groups each coordinate three Li^+^ cations, which gives rise to the infinite three-dimensional structure.

## Related literature

For Na salt synthesis, see: Lee & Kohn (1990 ▶). For Na salt, K salt, and acid form synthesis, see: Wen *et al.* (1975 ▶). 
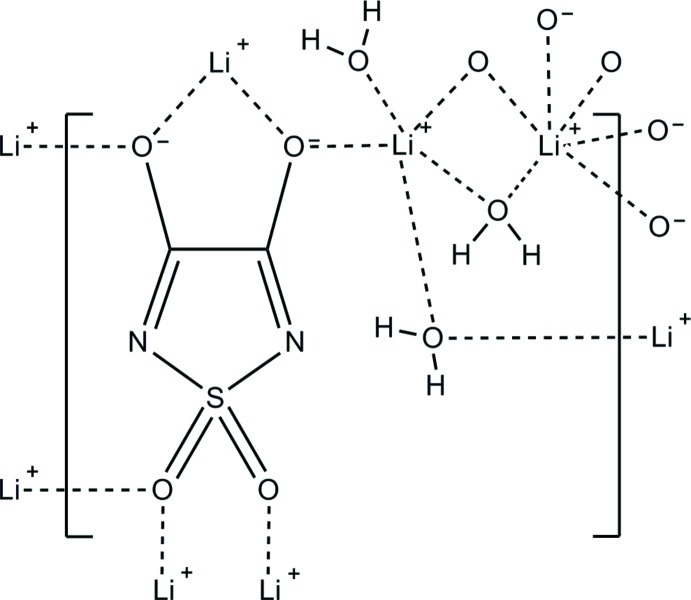


## Experimental

### 

#### Crystal data

[Li_2_(C_2_N_2_O_4_S)(H_2_O)_2_]
*M* *_r_* = 198.01
Monoclinic, 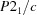

*a* = 7.239 (3) Å
*b* = 11.185 (3) Å
*c* = 9.786 (4) Å
β = 124.27 (2)°
*V* = 654.8 (4) Å^3^
*Z* = 4
Mo *K*α radiation
μ = 0.49 mm^−1^
*T* = 110 K
0.41 × 0.24 × 0.24 mm

#### Data collection

Bruker–Nonius Kappa X8 APEXII diffractometer
Absorption correction: multi-scan (*SADABS*; Bruker, 2009 ▶) *T*_min_ = 0.825, *T*_max_ = 0.894
34710 measured reflections
3899 independent reflections
3575 reflections with *I* > 2σ(*I*)
*R*_int_ = 0.022

#### Refinement

*R*[*F* ^2^ > 2σ(*F* ^2^)] = 0.022
*wR*(*F* ^2^) = 0.065
*S* = 1.06
3899 reflections
134 parameters
All H-atom parameters refined
Δρ_max_ = 0.62 e Å^−3^
Δρ_min_ = −0.40 e Å^−3^

### 

Data collection: *APEX2* (Bruker, 2009 ▶); cell refinement: *SAINT* (Bruker, 2009 ▶); data reduction: *SAINT*; program(s) used to solve structure: *SHELXS97* (Sheldrick, 2008 ▶); program(s) used to refine structure: *SHELXL97* (Sheldrick, 2008 ▶); molecular graphics: *ORTEP-3* (Farrugia, 1997 ▶); software used to prepare material for publication: *SHELXL97*.

## Supplementary Material

Crystal structure: contains datablock(s) I, global. DOI: 10.1107/S1600536812036379/qm2080sup1.cif

Structure factors: contains datablock(s) I. DOI: 10.1107/S1600536812036379/qm2080Isup2.hkl

Additional supplementary materials:  crystallographic information; 3D view; checkCIF report

## Figures and Tables

**Table 1:** Hydrogen-bond geometry (Å, °)

*D*—H⋯*A*	*D*—H	H⋯*A*	*D*⋯*A*	*D*—H⋯*A*
O1*W*—H1*WA*⋯N2^i^	0.791 (16)	2.155 (16)	2.9428 (14)	175 (2)
O1*W*—H1*WB*⋯O1^ii^	0.842 (17)	2.29 (2)	2.9971 (15)	142 (2)
O2*W*—H2*WA*⋯N2^iii^	0.823 (16)	2.049 (16)	2.8712 (14)	175.7 (16)
O2*W*—H2*WB*⋯N1^iv^	0.79 (2)	2.01 (2)	2.7947 (14)	168.7 (17)

Symmetry codes: (i) 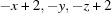; (ii) 
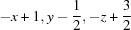
; (iii) 
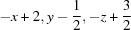
; (iv) 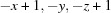.

**Fig. 1.**
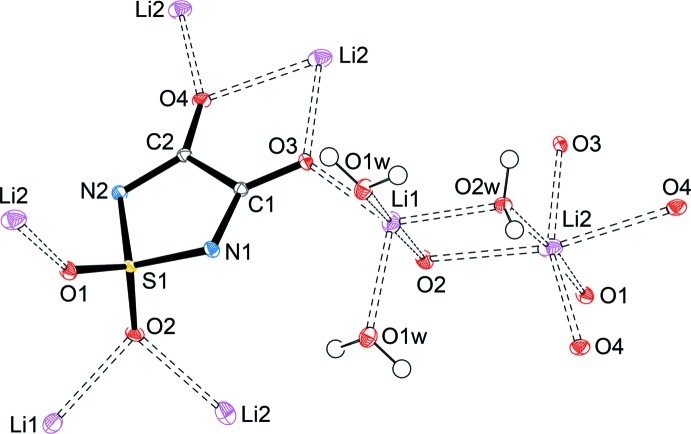
Asymmetric unit of the Li2TDD dihydrate crystal structure. Thermal ellipsoids are at 50% probability (Li-purple, O-red, S-yellow, N-blue).

**Fig. 2.**
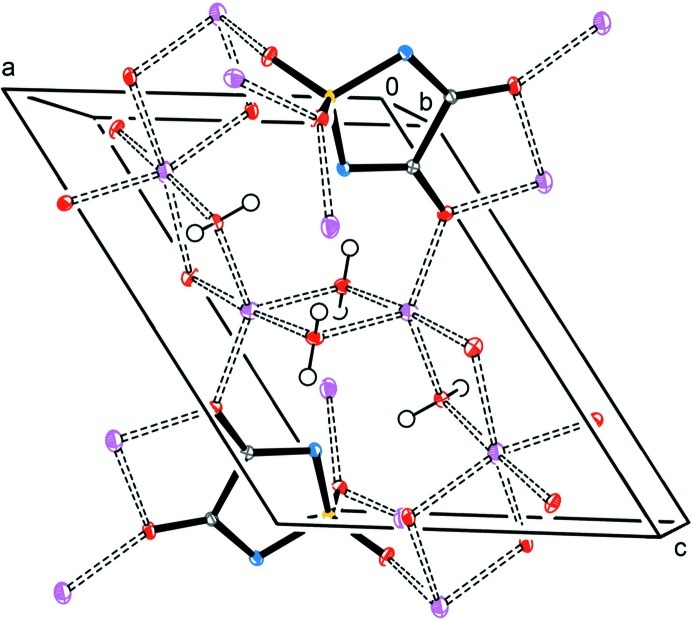
Packing diagram for the Li2TDD dihydrate crystal structure (Li-purple, O-red, S-yellow, N-blue).

## supplementary crystallographic information

### Comment

Dilithium 1,2,5-thiadiazolidine-3,4-dione 1,1-dioxide (Li_2_TDD) was synthesized following methods reported in the literature for the anion (Lee *et al.*, Wen *et al.*)*.* The salt forms a dihydrate in which the carbonyl and sulfuryl oxygen atoms each coordinate multiple Li^+^ cations forming a highly aggregated structure. In addition, each water molecule bridges two Li^+^ cations. The endocyclic nitrogen atoms, however, are not coordinated to the Li^+^ cations, indicating that the O atoms atoms exhibit the greatest Lewis basicity.

### Experimental

Sulfamide (1.00 eq.) was dissolved in methanol and slowly added to a solution of LiMeO (2.10 eq.) at room temperature, resulting in a cloudy white solution. After addition of diethyl oxalate (1.05 eq.), the solution became clear. After gently refluxing for 20 h, the solvent was removed under vacuum and a clear viscous solution was obtained. Upon addition of deionized water, crystals suitable for characterization formed.

### Refinement

The structure was solved by direct methods using the XS program. All non-hydrogen atoms were obtained from the i nitial solution. The hydrogen atoms were introduced at idealized positions and were allowed to refine isotropically. The structural model was fit to the data using full matrix least-squares based on F^2^. The calculated structure factors included corrections for anomalous dispersion from the usual tabulation. The structure was refined using the XL program from *SHELXTL*, graphic plots were produced using the *ORTEP-3* crystallographic program suite.

### Crystal data

**Table:**

[Li_2_(C_2_N_2_O_4_S)(H_2_O_2_)]	*F*(000) = 400
*M**_r_* = 198.01	*D*_x_ = 2.008 Mg m^−^^3^
Monoclinic, *P*2_1_/*c*	Mo *K*α radiation, λ = 0.71073 Å
*a* = 7.239 (3) Å	Cell parameters from 9851 reflections
*b* = 11.185 (3) Å	θ = 3.1–42.4°
*c* = 9.786 (4) Å	µ = 0.49 mm^−^^1^
β = 124.27 (2)°	*T* = 110 K
*V* = 654.8 (4) Å^3^	Prism, colourless
*Z* = 4	0.41 × 0.24 × 0.24 mm

### Data collection

**Table:**

Bruker–Nonius Kappa X8 APEXII diffractometer	3899 independent reflections
Radiation source: fine-focus sealed tube	3575 reflections with *I* > 2σ(*I*)
Graphite monochromator	*R*_int_ = 0.022
ω and φ scans	θ_max_ = 42.7°, θ_min_ = 3.1°
Absorption correction: multi-scan (*SADABS*; Bruker, 2009)	*h* = −13→12
*T*_min_ = 0.825, *T*_max_ = 0.894	*k* = −18→19
34710 measured reflections	*l* = −16→18

### Refinement

**Table:**

Refinement on *F*^2^	Primary atom site location: structure-invariant direct methods
Least-squares matrix: full	Secondary atom site location: difference Fourier map
*R*[*F*^2^ > 2σ(*F*^2^)] = 0.022	Hydrogen site location: inferred from neighbouring sites
*wR*(*F*^2^) = 0.065	All H-atom parameters refined
*S* = 1.06	*w* = 1/[σ^2^(*F*_o_^2^) + (0.0343*P*)^2^ + 0.1822*P*] where *P* = (*F*_o_^2^ + 2*F*_c_^2^)/3
3899 reflections	(Δ/σ)_max_ = 0.001
134 parameters	Δρ_max_ = 0.62 e Å^−^^3^
0 restraints	Δρ_min_ = −0.40 e Å^−^^3^

### Special details

**Table:**

Geometry. All e.s.d.'s (except the e.s.d. in the dihedral angle between two l.s. planes) are estimated using the full covariance matrix. The cell e.s.d.'s are taken into account individually in the estimation of e.s.d.'s in distances, angles and torsion angles; correlations between e.s.d.'s in cell parameters are only used when they are defined by crystal symmetry. An approximate (isotropic) treatment of cell e.s.d.'s is used for estimating e.s.d.'s involving l.s. planes.
Refinement. Refinement of *F*^2^ against ALL reflections. The weighted *R*-factor *wR* and goodness of fit *S* are based on *F*^2^, conventional *R*-factors *R* are based on *F*, with *F* set to zero for negative *F*^2^. The threshold expression of *F*^2^ > σ(*F*^2^) is used only for calculating *R*-factors(gt) *etc*. and is not relevant to the choice of reflections for refinement. *R*-factors based on *F*^2^ are statistically about twice as large as those based on *F*, and *R*– factors based on ALL data will be even larger.

### Fractional atomic coordinates and isotropic or equivalent isotropic displacement parameters (Å^2^)

**Table:**

	*x*	*y*	*z*	*U*_iso_*/*U*_eq_	
Li1	0.7137 (2)	0.01566 (12)	0.50356 (15)	0.0135 (2)	
Li2	0.7135 (2)	0.05125 (12)	0.18236 (17)	0.0149 (2)	
S1	0.89112 (2)	0.223295 (11)	0.983764 (16)	0.00633 (4)	
O1	0.79819 (8)	0.19195 (4)	1.07658 (6)	0.01045 (7)	
O2	0.84435 (8)	0.34610 (4)	0.92528 (6)	0.01025 (7)	
N1	0.79993 (8)	0.13338 (4)	0.82836 (6)	0.00815 (8)	
N2	1.16044 (8)	0.19981 (5)	1.09118 (6)	0.00814 (7)	
C1	0.97800 (9)	0.08981 (5)	0.83782 (7)	0.00681 (8)	
C2	1.19895 (9)	0.12853 (5)	0.99882 (7)	0.00704 (8)	
O3	0.97919 (7)	0.02518 (4)	0.73492 (5)	0.00883 (7)	
O4	1.38036 (7)	0.09387 (4)	1.02912 (6)	0.01080 (8)	
O1W	0.56092 (8)	−0.12147 (4)	0.56167 (6)	0.01060 (7)	
H1WA	0.642 (3)	−0.1436 (15)	0.654 (2)	0.038 (4)*	
H1WB	0.497 (3)	−0.1828 (15)	0.504 (2)	0.037 (4)*	
O2W	0.64205 (7)	−0.06718 (4)	0.30255 (6)	0.00860 (7)	
H2WA	0.702 (2)	−0.1334 (14)	0.3304 (18)	0.030 (3)*	
H2WB	0.512 (3)	−0.0804 (14)	0.2543 (19)	0.032 (4)*	

### Atomic displacement parameters (Å^2^)

**Table:**

	*U*^11^	*U*^22^	*U*^33^	*U*^12^	*U*^13^	*U*^23^
Li1	0.0127 (5)	0.0155 (5)	0.0110 (5)	−0.0009 (4)	0.0059 (4)	−0.0018 (4)
Li2	0.0120 (5)	0.0169 (5)	0.0176 (5)	0.0033 (4)	0.0094 (5)	0.0054 (4)
S1	0.00648 (6)	0.00602 (6)	0.00737 (6)	0.00044 (3)	0.00444 (5)	−0.00002 (3)
O1	0.01270 (18)	0.01092 (17)	0.01269 (18)	0.00098 (14)	0.01016 (16)	0.00139 (13)
O2	0.01357 (18)	0.00648 (15)	0.01303 (18)	0.00244 (13)	0.00890 (16)	0.00195 (13)
N1	0.00613 (17)	0.00947 (17)	0.00851 (17)	−0.00019 (13)	0.00391 (15)	−0.00189 (14)
N2	0.00659 (17)	0.00924 (17)	0.00772 (17)	−0.00007 (14)	0.00350 (15)	−0.00125 (14)
C1	0.00696 (19)	0.00660 (18)	0.00715 (18)	0.00008 (14)	0.00413 (16)	0.00011 (14)
C2	0.00620 (18)	0.00760 (18)	0.00714 (18)	0.00043 (14)	0.00365 (16)	0.00069 (14)
O3	0.00976 (17)	0.00902 (16)	0.00814 (16)	0.00060 (13)	0.00530 (14)	−0.00166 (12)
O4	0.00685 (16)	0.01436 (18)	0.01147 (17)	0.00300 (13)	0.00532 (15)	0.00209 (14)
O1W	0.01054 (17)	0.01104 (17)	0.00911 (17)	−0.00189 (13)	0.00487 (15)	−0.00023 (13)
O2W	0.00740 (16)	0.00840 (15)	0.00953 (16)	0.00014 (12)	0.00449 (14)	0.00025 (12)

### Geometric parameters (Å, º)

**Table:**

Li1—O2W	1.9615 (15)	S1—N2	1.6331 (9)
Li1—O3	1.9825 (16)	O1—Li2^vii^	2.1539 (14)
Li1—O1W^i^	2.0814 (16)	O2—Li1^viii^	2.1650 (14)
Li1—O1W	2.1468 (15)	O2—Li2^viii^	2.3088 (17)
Li1—O2^ii^	2.1650 (14)	N1—C1	1.3317 (9)
Li1—Li1^i^	3.076 (3)	N2—C2	1.3469 (8)
Li1—Li2	3.167 (2)	C1—O3	1.2435 (7)
Li1—H2WB	2.286 (16)	C1—C2	1.5460 (10)
Li2—O2W	2.0226 (14)	C2—O4	1.2346 (8)
Li2—O4^iii^	2.0539 (17)	O3—Li2^iv^	2.0755 (16)
Li2—O3^iv^	2.0755 (16)	O4—Li2^ix^	2.0539 (17)
Li2—O1^v^	2.1539 (14)	O4—Li2^iv^	2.4075 (17)
Li2—O2^ii^	2.3088 (17)	O1W—Li1^i^	2.0814 (16)
Li2—O4^iv^	2.4075 (17)	O1W—H1WA	0.792 (17)
Li2—Li2^vi^	3.344 (3)	O1W—H1WB	0.840 (17)
S1—O1	1.4453 (6)	O2W—H2WA	0.824 (16)
S1—O2	1.4529 (6)	O2W—H2WB	0.794 (16)
S1—N1	1.6216 (7)		
			
O2W—Li1—O3	135.20 (8)	O1^v^—Li2—Li1	136.68 (7)
O2W—Li1—O1W^i^	107.07 (7)	O2^ii^—Li2—Li1	43.12 (4)
O3—Li1—O1W^i^	117.64 (7)	O4^iv^—Li2—Li1	127.63 (6)
O2W—Li1—O1W	92.49 (6)	O2W—Li2—Li2^vi^	90.62 (7)
O3—Li1—O1W	92.78 (6)	O4^iii^—Li2—Li2^vi^	45.64 (4)
O1W^i^—Li1—O1W	86.66 (6)	O3^iv^—Li2—Li2^vi^	114.74 (8)
O2W—Li1—O2^ii^	84.50 (6)	O1^v^—Li2—Li2^vi^	94.57 (7)
O3—Li1—O2^ii^	91.73 (6)	O2^ii^—Li2—Li2^vi^	150.02 (7)
O1W^i^—Li1—O2^ii^	91.01 (6)	O4^iv^—Li2—Li2^vi^	37.58 (4)
O1W—Li1—O2^ii^	175.48 (7)	Li1—Li2—Li2^vi^	119.99 (7)
O2W—Li1—Li1^i^	103.23 (7)	O1—S1—O2	112.91 (3)
O3—Li1—Li1^i^	110.35 (8)	O1—S1—N1	110.57 (4)
O1W^i^—Li1—Li1^i^	44.16 (4)	O2—S1—N1	109.60 (4)
O1W—Li1—Li1^i^	42.49 (4)	O1—S1—N2	111.47 (4)
O2^ii^—Li1—Li1^i^	135.04 (8)	O2—S1—N2	109.54 (3)
O2W—Li1—Li2	38.02 (4)	N1—S1—N2	102.23 (3)
O3—Li1—Li2	125.81 (7)	S1—O1—Li2^vii^	146.99 (5)
O1W^i^—Li1—Li2	99.07 (6)	S1—O2—Li1^viii^	125.95 (5)
O1W—Li1—Li2	129.81 (6)	S1—O2—Li2^viii^	138.18 (4)
O2^ii^—Li1—Li2	46.80 (4)	Li1^viii^—O2—Li2^viii^	90.08 (5)
Li1^i^—Li1—Li2	123.61 (7)	C1—N1—S1	107.15 (5)
O2W—Li1—H2WB	19.7 (4)	C2—N2—S1	106.65 (5)
O3—Li1—H2WB	150.4 (4)	O3—C1—N1	127.18 (6)
O1W^i^—Li1—H2WB	90.8 (4)	O3—C1—C2	120.88 (5)
O1W—Li1—H2WB	80.3 (4)	N1—C1—C2	111.95 (5)
O2^ii^—Li1—H2WB	95.9 (4)	O4—C2—N2	128.23 (6)
Li1^i^—Li1—H2WB	83.8 (4)	O4—C2—C1	120.46 (6)
Li2—Li1—H2WB	50.0 (4)	N2—C2—C1	111.31 (5)
O2W—Li2—O4^iii^	89.83 (6)	C1—O3—Li1	120.78 (6)
O2W—Li2—O3^iv^	94.15 (6)	C1—O3—Li2^iv^	114.73 (6)
O4^iii^—Li2—O3^iv^	160.11 (8)	Li1—O3—Li2^iv^	124.06 (6)
O2W—Li2—O1^v^	173.35 (8)	C2—O4—Li2^ix^	154.29 (6)
O4^iii^—Li2—O1^v^	90.83 (6)	C2—O4—Li2^iv^	104.96 (5)
O3^iv^—Li2—O1^v^	87.46 (5)	Li2^ix^—O4—Li2^iv^	96.78 (6)
O2W—Li2—O2^ii^	79.51 (6)	Li1^i^—O1W—Li1	93.34 (6)
O4^iii^—Li2—O2^ii^	105.58 (6)	Li1^i^—O1W—H1WA	119.4 (12)
O3^iv^—Li2—O2^ii^	94.31 (6)	Li1—O1W—H1WA	111.6 (12)
O1^v^—Li2—O2^ii^	93.94 (6)	Li1^i^—O1W—H1WB	100.8 (11)
O2W—Li2—O4^iv^	91.01 (6)	Li1—O1W—H1WB	125.3 (11)
O4^iii^—Li2—O4^iv^	83.22 (6)	H1WA—O1W—H1WB	106.4 (16)
O3^iv^—Li2—O4^iv^	77.24 (5)	Li1—O2W—Li2	105.30 (7)
O1^v^—Li2—O4^iv^	95.64 (6)	Li1—O2W—H2WA	107.5 (10)
O2^ii^—Li2—O4^iv^	166.89 (6)	Li2—O2W—H2WA	121.4 (10)
O2W—Li2—Li1	36.68 (4)	Li1—O2W—H2WB	103.9 (11)
O4^iii^—Li2—Li1	95.63 (6)	Li2—O2W—H2WB	112.5 (11)
O3^iv^—Li2—Li1	99.19 (6)	H2WA—O2W—H2WB	104.9 (15)
			
O3—Li1—Li2—O2W	119.85 (9)	O2—S1—N1—C1	107.86 (4)
O1W^i^—Li1—Li2—O2W	−106.16 (7)	N2—S1—N1—C1	−8.27 (4)
O1W—Li1—Li2—O2W	−12.94 (6)	O1—S1—N2—C2	125.58 (5)
O2^ii^—Li1—Li2—O2W	171.05 (8)	O2—S1—N2—C2	−108.72 (5)
Li1^i^—Li1—Li2—O2W	−66.17 (8)	N1—S1—N2—C2	7.45 (4)
O2W—Li1—Li2—O4^iii^	82.11 (7)	S1—N1—C1—O3	−173.88 (5)
O3—Li1—Li2—O4^iii^	−158.04 (7)	S1—N1—C1—C2	6.29 (5)
O1W^i^—Li1—Li2—O4^iii^	−24.05 (6)	S1—N2—C2—O4	175.91 (5)
O1W—Li1—Li2—O4^iii^	69.17 (9)	S1—N2—C2—C1	−4.32 (5)
O2^ii^—Li1—Li2—O4^iii^	−106.84 (7)	O3—C1—C2—O4	−1.36 (8)
Li1^i^—Li1—Li2—O4^iii^	15.94 (10)	N1—C1—C2—O4	178.47 (5)
O2W—Li1—Li2—O3^iv^	−84.58 (7)	O3—C1—C2—N2	178.85 (5)
O3—Li1—Li2—O3^iv^	35.28 (9)	N1—C1—C2—N2	−1.32 (6)
O1W^i^—Li1—Li2—O3^iv^	169.26 (6)	N1—C1—O3—Li1	19.12 (9)
O1W—Li1—Li2—O3^iv^	−97.52 (9)	C2—C1—O3—Li1	−161.08 (6)
O2^ii^—Li1—Li2—O3^iv^	86.47 (6)	N1—C1—O3—Li2^iv^	−168.10 (6)
Li1^i^—Li1—Li2—O3^iv^	−150.75 (8)	C2—C1—O3—Li2^iv^	11.71 (7)
O2W—Li1—Li2—O1^v^	179.33 (11)	O2W—Li1—O3—C1	177.90 (8)
O3—Li1—Li2—O1^v^	−60.82 (12)	O1W^i^—Li1—O3—C1	1.93 (10)
O1W^i^—Li1—Li2—O1^v^	73.17 (10)	O1W—Li1—O3—C1	−85.75 (7)
O1W—Li1—Li2—O1^v^	166.39 (7)	O2^ii^—Li1—O3—C1	93.97 (6)
O2^ii^—Li1—Li2—O1^v^	−9.62 (8)	Li1^i^—Li1—O3—C1	−46.05 (10)
Li1^i^—Li1—Li2—O1^v^	113.16 (10)	Li2—Li1—O3—C1	128.60 (7)
O2W—Li1—Li2—O2^ii^	−171.05 (8)	O2W—Li1—O3—Li2^iv^	5.82 (13)
O3—Li1—Li2—O2^ii^	−51.20 (7)	O1W^i^—Li1—O3—Li2^iv^	−170.16 (6)
O1W^i^—Li1—Li2—O2^ii^	82.79 (6)	O1W—Li1—O3—Li2^iv^	102.16 (7)
O1W—Li1—Li2—O2^ii^	176.01 (9)	O2^ii^—Li1—O3—Li2^iv^	−78.11 (7)
Li1^i^—Li1—Li2—O2^ii^	122.78 (10)	Li1^i^—Li1—O3—Li2^iv^	141.87 (7)
O2W—Li1—Li2—O4^iv^	−3.65 (6)	Li2—Li1—O3—Li2^iv^	−43.48 (11)
O3—Li1—Li2—O4^iv^	116.20 (8)	N2—C2—O4—Li2^ix^	25.13 (16)
O1W^i^—Li1—Li2—O4^iv^	−109.82 (7)	C1—C2—O4—Li2^ix^	−154.62 (11)
O1W—Li1—Li2—O4^iv^	−16.60 (11)	N2—C2—O4—Li2^iv^	171.92 (6)
O2^ii^—Li1—Li2—O4^iv^	167.39 (8)	C1—C2—O4—Li2^iv^	−7.83 (7)
Li1^i^—Li1—Li2—O4^iv^	−69.83 (10)	O2W—Li1—O1W—Li1^i^	−106.96 (7)
O2W—Li1—Li2—Li2^vi^	41.08 (7)	O3—Li1—O1W—Li1^i^	117.54 (7)
O3—Li1—Li2—Li2^vi^	160.93 (7)	O1W^i^—Li1—O1W—Li1^i^	0.0
O1W^i^—Li1—Li2—Li2^vi^	−65.09 (9)	O2^ii^—Li1—O1W—Li1^i^	−58.9 (9)
O1W—Li1—Li2—Li2^vi^	28.13 (11)	Li2—Li1—O1W—Li1^i^	−99.03 (8)
O2^ii^—Li1—Li2—Li2^vi^	−147.88 (9)	O3—Li1—O2W—Li2	−93.45 (11)
Li1^i^—Li1—Li2—Li2^vi^	−25.09 (12)	O1W^i^—Li1—O2W—Li2	82.82 (7)
O2—S1—O1—Li2^vii^	166.26 (8)	O1W—Li1—O2W—Li2	170.08 (5)
N1—S1—O1—Li2^vii^	43.06 (9)	O2^ii^—Li1—O2W—Li2	−6.54 (6)
N2—S1—O1—Li2^vii^	−69.93 (9)	Li1^i^—Li1—O2W—Li2	128.50 (7)
O1—S1—O2—Li1^viii^	12.38 (6)	O4^iii^—Li2—O2W—Li1	−99.68 (6)
N1—S1—O2—Li1^viii^	136.11 (6)	O3^iv^—Li2—O2W—Li1	99.83 (7)
N2—S1—O2—Li1^viii^	−112.48 (6)	O1^v^—Li2—O2W—Li1	−4.0 (6)
O1—S1—O2—Li2^viii^	−132.04 (7)	O2^ii^—Li2—O2W—Li1	6.21 (6)
N1—S1—O2—Li2^viii^	−8.31 (7)	O4^iv^—Li2—O2W—Li1	177.11 (5)
N2—S1—O2—Li2^viii^	103.09 (7)	Li2^vi^—Li2—O2W—Li1	−145.31 (6)
O1—S1—N1—C1	−127.04 (4)		

Symmetry codes: (i) −*x*+1, −*y*, −*z*+1; (ii) *x*, −*y*+1/2, *z*−1/2; (iii) *x*−1, *y*, *z*−1; (iv) −*x*+2, −*y*, −*z*+1; (v) *x*, *y*, *z*−1; (vi) −*x*+1, −*y*, −*z*; (vii) *x*, *y*, *z*+1; (viii) *x*, −*y*+1/2, *z*+1/2; (ix) *x*+1, *y*, *z*+1.

### Hydrogen-bond geometry (Å, º)

**Table:**

*D*—H···*A*	*D*—H	H···*A*	*D*···*A*	*D*—H···*A*
O1*W*—H1*WA*···N2^x^	0.791 (16)	2.155 (16)	2.9428 (14)	175 (2)
O1*W*—H1*WB*···O1^xi^	0.842 (17)	2.29 (2)	2.9971 (15)	142 (2)
O2*W*—H2*WA*···N2^xii^	0.823 (16)	2.049 (16)	2.8712 (14)	175.7 (16)
O2*W*—H2*WB*···N1^i^	0.79 (2)	2.01 (2)	2.7947 (14)	168.7 (17)

Symmetry codes: (i) −*x*+1, −*y*, −*z*+1; (x) −*x*+2, −*y*, −*z*+2; (xi) −*x*+1, *y*−1/2, −*z*+3/2; (xii) −*x*+2, *y*−1/2, −*z*+3/2.
